# VDAC1 regulates neuronal cell loss after retinal trauma injury by a mitochondria-independent pathway

**DOI:** 10.1038/s41419-022-04755-3

**Published:** 2022-04-21

**Authors:** Erica de Sousa, Marília Inês Móvio, Théo Henrique de Lima-Vasconcellos, Gabrieli Bovi dos Santos, Talita dos Santos Gomes, Lais Takata Walter, Daniela Almeida da Silva, Tiago Rodrigues, Giselle Cerchiaro, Alexandre Hiroaki Kihara

**Affiliations:** 1 Neurogenetics Laboratory, Centro de Matemática, Computação e Cognição, Universidade Federal do ABC, São Bernardo do Campo, SP Brazil; 2 Metal Biochemistry and Oxidative Stress Laboratory, Centro de Ciências Naturais e Humanas, Universidade Federal do ABC, Santo André, SP Brazil; 3 Centro de Ciências Naturais e Humanas, Universidade Federal do ABC, Santo André, SP Brazil

**Keywords:** Cell death in the nervous system, Cellular neuroscience

## Abstract

The voltage-dependent anion channel 1 (VDAC1) was first described as a mitochondrial porin that mediates the flux of metabolites and ions, thereby integrating both cell survival and death signals. In the nervous system, the functional roles of VDAC1 remain poorly understood. Herein, the rat retina was employed to study VDAC1. First, it was observed that even subtle changes in VDAC1 levels affect neuronal survival, inducing severe alterations in the retinal morphology. We next examined the regulation of VDAC1 after traumatic retinal injury. After mechanical trauma, SOD1 translocates towards the nucleus, which is insufficient to contain the consequences of oxidative stress, as determined by the evaluation of protein carbonylation. Using in vitro models of oxidative stress and mechanical injury in primary retinal cell cultures, it was possible to determine that inhibition of VDAC1 oligomerization by 4′-diisothiocyano-2,2′-disulfonic acid stilbene (DIDS) rescues cell viability, impacting microglial cell activation. We next focused on the regulation of VDAC1 after retinal mechanical injury. VDAC1 was promptly upregulated 2 h after lesion in the plasma membrane and endoplasmic reticulum rather than in the mitochondria, and multimers of VDAC1 were assembled after lesion. DIDS intraocular application decreased apoptosis and prevented microglial polarization, which confirmed in vitro observations. Considering the role of microglia in neuroinflammation, multiplex evaluation of cytokines showed that DIDS application disorganized the inflammatory response 2 h after the lesion, matching the fast regulation of VDAC1. Taken together, data disclosed that fine regulation of VDAC1 influences neuronal survival, and pharmacological inhibition after trauma injury has neuroprotective effects. This protection may be attributed to the effects on VDAC1 abnormal accumulation in the plasma membrane, thereby controlling the activation of microglial cells. We concluded that VDAC1 is a putative therapeutic target in neuronal disorders since it integrates both death and survival cellular signaling.

## Introduction

Among the pathological conditions of the nervous system, primary degeneration encompasses the death of cells directly exposed to a harmful stimulus. The affected cells release toxic factors that compromise neighbor cells, a process known as secondary degeneration, which occurs after traumatic injuries [1, 2] and the onset and progression of neurodegenerative diseases [3]. Calcium dysregulation [4, 5], reactive oxygen species [6, 7], inflammation [8], and glutamatergic toxicity [9], among others, are triggers of secondary cell death.

Several proteins are recruited to promote cell death, including the voltage-dependent ion channel 1 (VDAC1). VDAC1 allows the passage of molecules up to 5 kDa, including ions, nucleotides, and other metabolites across the outer mitochondrial membrane [10]. VDAC1 channel is a pathway to mitochondrial translocation of α-synuclein, a mechanism related to the loss of dopaminergic neurons in Parkinson’s disease [11]. In the mouse model of amyotrophic lateral sclerosis, a misfolded mutant of superoxide dismutase 1 (SOD1) directly inhibits VDAC1 conductance [12, 13], disclosing the connection of VDAC1 and SOD1 in oxidative stress [14, 15]. VDAC1 can also be located in other subcellular compartments, where its role remains under discussion [16, 17], since independent groups found VDAC1 in the plasma membrane [18–20] and endoplasmic reticulum (ER) [21]. VDAC1 excess has been observed in pathologies of the nervous system associated with cell loss [22–24], such as in the brains of post-mortem AD patients, AD-like transgenic mice [25, 26], and other neurodegenerative disease models [27].

VDAC1 has been implicated in neuronal cell survival and death mechanisms. In the high-conductance state, the channel is permeable to organic anions, including respiratory substrates, ATP, and ADP, whereas in the low-conductance state, the channel transports K^+^, Na^+^, and Ca^2+^, thereby playing essential roles in the outer mitochondrial metabolism [28]. The traffic of larger molecules is often associated with VDAC1 oligomerization, a process that can be prevented with 4,4′-diisothiocyano-2,2′-stilbenedisulfonic acid (DIDS) [29–31]. Herein, we aimed to evaluate the role of VDAC1 in the healthy and degenerating retina after mechanical injury, a process associated with oxidative stress.

## Results

### VDAC1 plays an essential role in the maintenance of retinal morphology, and its knockdown is harmful to specific neuronal subtypes

**Fig. 1:**
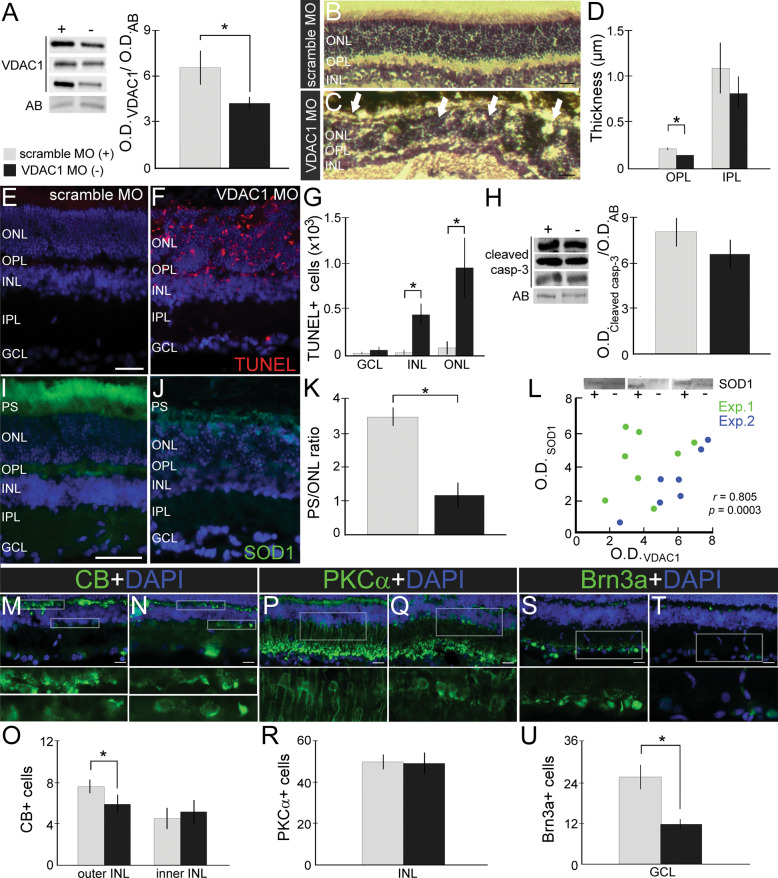
Voltage-dependent anion channel 1 (VDAC1) knockdown induces morphological alterations and cell death in the retina, which can be associated with the impact on specific retinal cell subtypes. (**A**) VDAC1 protein levels after scramble morpholino (+) and VDAC1 oligonucleotide antisense morpholino (−) interventions. Representative bands of VDAC1 and amido black (AB) in Western blot experiments, and quantification of normalized VDAC1 optic density (OD) (*n* = 6). Representative images of transverse sections from (**B**) scramble MO and (**C**) VDAC1 MO treated retinas stained with hematoxylin and eosin. White arrows indicate rosette formation in the outer nuclear layer (ONL). **D** Thickness of outer plexiform layer (OPL) and inner plexiform layer (IPL) after scramble MO and VDAC1 MO treatment (*n* = 5). Representative images of transverse sections from (**E**) scramble MO and (**F**) VDAC1 MO treated retinas stained with TUNEL (red) and counterstained with 4′,6-diamidino-2-phenylindole (DAPI, blue). **G** Quantification of TUNEL-positive cells in the retinal nuclear layers (*n* = 5). **H** Representative bands of cleaved caspase-3 and AB in Western blot experiments and quantification of normalized cleaved caspase-3 OD (*n* = 8). **I**–**J** Superoxide dismutase 1 (SOD1) protein immunofluorescence (IF, green) in the scramble MO and VDAC1 MO treated retinas. **K** Photoreceptor’s segment (PS) and outer nuclear layer (ONL) labeling ratio in scramble MO and VDAC1 MO retinas (*n* = 4). (L) Pearson’s correlation analysis of SOD1 in VDAC1 knockdown experiments. Up: Representative bands of SOD1 in western blot experiments; down: correlation graph showing the OD of SOD1 and VDAC1 in experiment 1 (Exp.1, green) and experiment 2 (Exp.2, blue). The Pearson coefficient *r* and *p* value (*n* = 8) are indicated in the graph. **M**, **N** Calbindin (CB) immunofluorescence (IF, green) in scramble MO and VDAC1 MO treated retinas with high magnification of the selected area in OPL [65] and inner nuclear layer (INL, down). **O** Quantification of CB-positive cells after treatment (*n* = 5). **P**, **Q** PKC-alpha (PKCα) IF (green) in scramble MO and VDAC1 MO treated retinas with high magnification of the selected areas in INL and IPL. **R** Quantification of PKCα-positive cells after treatment (*n* = 5). **S**–**T** Brn3a IF (green) in scramble MO and VDAC1 MO treated retinas with high magnification of the selected area in the ganglion cell layer (GCL). **U** Quantification of Brn3a-positive cells after treatment (*n* = 5). Data are represented as mean ± standard error of the mean. Scale bar: 25 μm. *P < 0.05 in paired Student’s *T*-test (**A**, **G**, **H**, **O**, **R**, **U**) or two-way ANOVA followed by Sidak’s post-hoc (**D**).

To understand the importance of VDAC1 in the retina, we first induced in vivo knockdown of VDAC1 using MO, in which a decrease of 44.87% of the protein levels was observed after 5 days (6.51 ± 1.16 vs. 3.59 ± 0.35, *P* < 0.05) (Fig. 1A). This reduction triggered structural alterations in the retina (Fig. 1B, C), which was more evident in the outer nuclear layer (ONL), outer plexiform layer (OPL), and inner nuclear layer (INL). It was possible to observe the photoreceptor segments (PS) pointing to the center of oval structures resembling rosettes. VDAC1 MO treated retinas presented a decrease in OPL (0.21 ± 0.01 vs. 0.15 ± 0.01, *P* < 0.05) but not in IPL (*P* > 0.05) thickness (Fig. 1D). TUNEL assay was employed after VDAC1 MO intervention (Fig. 1E, F). A significant increase of TUNEL-positive cells was observed in ONL (8.10 ± 7.33 vs. 94.69 ± 32.79, *p* < 0.05) and INL (2.00 ± 1.79 vs. 36.08 ± 9.93, *p* < 0.05), but not GCL (Fig. 1G). The apoptosis and the structural rearrangement promoted by VDAC1 knockdown seemed to be caspase-independent since cleaved caspase-3 levels did not exhibit significant alterations (*P* > 0.05) (Fig. 1H).

We focused on the distribution of SOD1 in VDAC1 MO retinas since the connection of these proteins was previously determined [12, 13]. In controls, SOD1 labeling was mainly observed in PS, while a diffuse pattern was detected in VDAC1 MO-treated retinas (Fig. 1I, J). A decrease in the PS/ONL labeling ratio was determined in MO VDAC1 treated retinas (3.49 ± 0.31 vs. 1.14 ± 0.39, *P* < 0.05) (Fig. 1K). To evaluate the impact of VDAC1 downregulation in SOD1 levels, we performed a Pearson correlation analysis using the respective O.D., which returned a very high positive correlation (*r* = 0.805, *P* < 0.001, Fig. 1L).

We next evaluated whether the VDAC1 knockdown could affect specific retinal cells other than the photoreceptors. The immunofluorescence (IF) analysis showed a decrease in calbindin (CB)-positive cells located in the outer INL in VDAC1 MO retinas (7.50 ± 0.66 vs. 5.90 ± 0.62, *P* < 0.05), but not in the inner INL (*P* > 0.05) (Fig. 1M–O). The knockdown of VDAC1 did not change the number of PKCα-positive bipolar cells (*P* > 0.05) (Fig. 1P, R), whereas a decrease in Brn3a-positive ganglion cells was observed (25.63 ± 3.60 vs. 11.87 ± 1.53, *P* < 0.05) (Fig. 1S–U).

### Mechanical trauma promotes nuclear translocation of SOD1 and increases protein carbonylation in the retina

Our results revealed that the retinal degeneration triggered by VDAC1 knockdown could be related to oxidative stress followed by caspase-independent apoptosis [32, 33]. Since we observed a significant correlation between VDAC1 and SOD1 levels and considering the prominent role of VDAC1 in oxidative stress, we analyzed the distribution of SOD1 using a model of mechanical trauma.

**Fig. 2:**
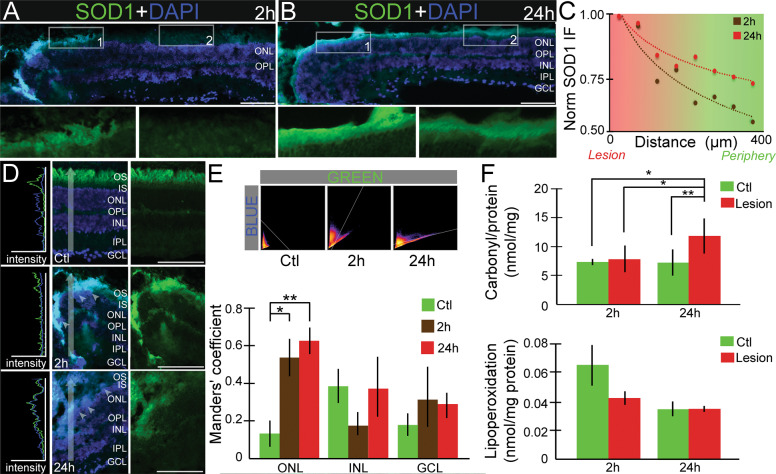
Mechanical trauma induces the SOD1 nuclear translocation increase protein carbonylation. Superoxide dismutase 1 (SOD1) protein immunofluorescence (IF, green) in the retina 2 h (**A**) and 24 h (**B**) after lesion, counterstained with 4–6-diamidino-2-phenylindole (DAPI, blue), with high magnification of photoreceptor segments. The arrows indicate SOD1 labeling in the segments. (**C**) Plot of normalized SOD1 intensity according to lesion distance, 2 h (brown) and 24 h (red) after lesion. The dotted lines indicate the logarithmic trend line (*n* = 5). **D** Representative IF of SOD1 and respective pixel profiles in controls and lesioned retinas. The vertical arrow indicates the analyzed area of the pixel profile. The white arrowheads indicate the nuclear SOD1 labeling. **E** Manders’ coefficient analysis of SOD1 and DAPI correlation. 2D histograms show a correlation in Ctl, 2 and 24 h, y-axis: DAPI [66] and *x*-axis: SOD1 (green). Manders’ coefficient analysis of SOD1/DAPI in ONL, INL, and GCL layers (*n* = 4). (**F**) Protein carbonylation and lipoperoxidation 2 and 24 h after lesion (*n* = 3). Data are presented as mean ± standard error of the mean. Scale bar: 50 μm. *P < 0.05 and **P < 0.01 in one-way ANOVA (**F**) and two-way ANOVA (**E**) followed by Sidak’s post-test. The layers indicate the approximate localization of the outer photoreceptor segments (OS), inner photoreceptor segments (IS), outer nuclear layer (ONL), outer plexiform layer (OPL), inner nuclear layer (INL), inner plexiform layer (IPL) and ganglion cell layer (GCL).

SOD1 accumulation in the lesion was observed as early as 2 h after mechanical trauma (Fig. 2A, B), especially in the inner PSs. SOD1 labeling decreased with the distance from the lesion (Fig. 2C), translocating towards the nucleus in the ONL (Fig. 2D). Manders’ colocalization analyses confirmed statistical differences in two-way ANOVA (F(4,24) = 2.795, *P* < 0.05). Simple main effect confirmed that SOD1 translocates to the nucleus 2 and 24 h after the lesion in ONL (Ctl: 0.13 ± 0.08; 2 h: 0.53 ± 0.13; 24 h: 0.62 ± 0.09; Ctl vs. 2 h, *P* < 0.05, Ctl vs. 24 h: *P* < 0.01), but not in INL or GCL (Fig. 2E).

There were no significant differences in the lipoperoxidation or carbonylation levels 2 h after lesion (*P* > 0.05). After 24 h the lesioned retinas exhibited increased levels of protein carbonylation compared to control (*P* < 0.01) or 2 h (*P* < 0.05) (Fig. 2F). ROS can more easily target proteins throughout the retina by accumulation, leading to permanent damage that is not repairable. This way, it is considered a sensitive biomarker for tracking oxidative stress.

### In vitro inhibition of VDAC1 generates neuroprotection against chemical or mechanical injury and impacts on microglial cell and astrocyte activation

We next evaluated the possible effects of VDAC1 pharmacological inhibitor (DIDS) using two in vitro models, H_2_O_2_-induced oxidative stress and mechanical scratch of the cell monolayer.

**Fig. 3:**
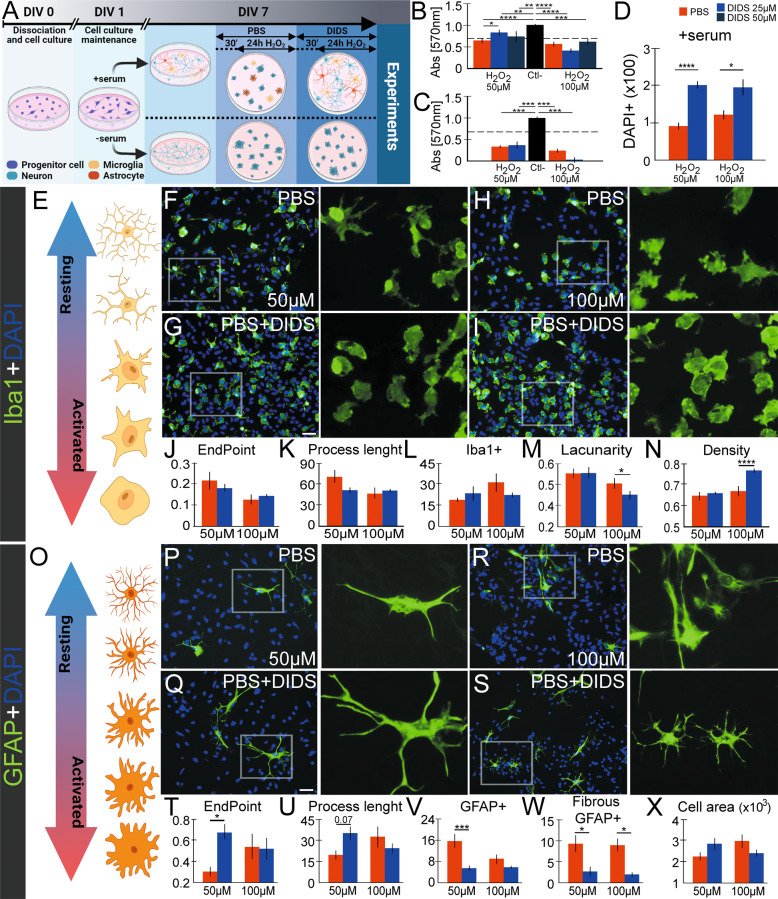
Effects of VDAC1 inhibitor 4,4′-diisothiocyano-2,2′-stilbenedisulfonic acid (DIDS) on in vitro model of oxidative stress. **A** General scheme of experiments, showing the treatment timeline and specific conditions. **B** The viability of mixed primary cell cultures treated with PBS (red) or DIDS (25 µM, blue) after H_2_O_2_ exposition (50 and 100 µM) as evaluated with MTT assay. The negative control (considered as 100% of viability) is indicated with the black bar (*n* = 6–10). **C** The viability of pure neuronal primary cell cultures treated with PBS (red) or DIDS (25 µM, blue) after H_2_O_2_ exposition (50 and 100 µM) as evaluated with MTT. The negative control (100% viability) is indicated with the back bar (*n* = 6–8). **D** Quantification of DAPI + nuclei in PBS and DIDS treatment after 50 µM H_2_O_2_ (left) and 100 µM H_2_O_2_ (right) (*n* = 9). **E** Representative illustration indicating the resting (up, represented in blue arrow) and active (down, red) microglial status. Iba1 IF (green) in PBS (**F**, **H**) and DIDS (**G**, **I**) treatment, both in 50 (**F**, **G**) and 100 µM (**H**, **I**) of H_2_O_2_. The digital magnificence in each image represents the indicated cell. Representative graphs of microglia ramifications, showing the normalized Iba1 endpoint per cell (**J**) and the mean of process length per cell (**K**). Both graphs represent *n* = 11–17 (L) Graph of Iba1 heterogeneity considering microglia lacunarity (*n* = 153–169). Quantification of Iba1+ cells distribution, considering Iba1 density (*M*, *n* = 153–169) and number of cells (*N*, *n* = 11–15). **O** Representative illustration indicating the resting (up, represented in blue arrow) and active (down, red) astrocyte status [67]. GFAP IF (green) in PBS (**P**, **R**) and DIDS (**Q**, **S**) treatment, both in 50 (**P**, **Q**) and 100 µM (**R**, **S**) of H_2_O_2_. The digital magnificence in each image represents the indicated cell. Representative graphs of astrocyte ramifications, showing the normalized GFAP endpoint per cell (**T**) and the mean of process length per cell (**U**). In both graphs *n* = 9–14. Quantification of GFAP cell distribution and morphology, considering total GFAP+ cells (**V**, *n* = 24–32), GFAP labeling, which shows a fibrous-like morphology (**W**, *n* = 24–32), and GFAP+ cell area (**X**, *n* = 16–25). The IF sections were counterstained with 4–6-diamidino-2-phenylindole (DAPI, blue). DIV: days in vitro *P < 0.05; **P < 0.01; ***P < 0.001; ****P < 0.0001 as indicated in one- (**B**–**C**, **L**–**M**, **V**–**W**) or two-way ANOVA (**D**, **J**–**K**, **N**, **T**–**U**, **X**) followed by Sidak’s post-hoc, except in MTT analysis, in which Fisher’s LSD post-hoc was employed. The illustrations were obtained using BioRender and Photoshop software.

Mixed primary cell cultures were obtained, and DIDS was pre-incubated 30 min before H_2_O_2_ exposition (Fig. 3A). The MTT results revealed that 50 and 100 µM H_2_O_2_ decreased the cell viability in primary cell cultures (63.66 ± 6.74% and 58.07 ± 6.74%, respectively). 25 µM DIDS, but not 50 µM, showed a viability over 75% in cells exposed to 50 µM H_2_O_2_ (25 µM DIDS + H_2_O_2_: 83.91 ± 6.81%, *P* < 0.05 vs. 50 µM H_2_O_2_; 50 µM DIDS 72.97 ± 14.48%, *P* > 0.05 vs. 50 µM H_2_O_2_) (Fig. 3B**)**. 25 µM and 50 µM DIDS did not rescue cell viability when cell cultures were exposed to 100 µM H_2_O_2_. We also evaluated the effects of DIDS in pure primary neuronal cell cultures. These cells were more susceptible to the H_2_O_2_ than the mixed primary cells, and DIDS did not preserve cell viability when exposed to H_2_O_2_ (*P* > 0.05, Fig. 3C), thereby evidencing the role of glial cells in this protection.

Since DIDS presented a protective effect only on mixed primary cell cultures, we employed this culture in the following experiments. DIDS increased the number of DAPI+ in cell cultures exposed to 50 µM H_2_O_2_ (90.33 ± 9.58 vs. 198.73 ± 13.98 cells, *P* < 0.001), and to 100 µM H_2_O_2_ (120.22 ± 16.92 vs. 193.40 ± 24.39, *P* < 0.05) (Fig. 3B). Since protective effects provided by DIDS were attributed to the presence of glial cells, we next analyzed microglial cells and astrocytes. Analysis of Iba1 did not reveal changes in the microglial morphology, ramification, heterogeneity or distribution in DIDS+ 50 µM H_2_O_2_ (*P* > 0.05). Significant alterations were observed in DIDS + 100 µM H_2_O_2_. DIDS increased the heterogeneity of microglial cells (lacunarity, 0.49 ± 0.01 vs. 0.44 ± 0.01, *P* < 0.05, Fig. 3M) and density (0.66 ± 0.01 vs. 0.74 ± 0.01, *P* < 0.001, Fig. 3N). The ramification process was not altered in both conditions (Fig. 3J, K), nor the number of Iba+ cells (Fig. 3L).

Anti-GFAP was employed to evaluate changes in astrocytes. DIDS in 50 µM H_2_O_2_, but not in 100 µM H_2_O_2_, increased complexity of astrocyte ramification (EndPoint: 0.30 ± 0.05 vs. 0.66 ± 0.09, *P* < 0.05, Fig. 3T) and generated a tendency towards an increase in the process length (19.07 ± 3.35 vs. 34.87 ± 5.22, *P* = 0.07, Fig. 3U). DIDS treatment decreased the number of GFAP + cells (15.43 ± 2.76 vs. 5.32 ± 1.24, *P* < 0.001, Fig. 3V) as well as the number of GFAP + with fibrous-like morphology in 50 µM (9.14 ± 2.42 vs. 2.56 ± 1.29, *P* < 0.05) and 100 µM H_2_O_2_ (9.00 ± 1.60 vs. 1.88 ± 0.42, *P* < 0.05, Fig. 3W). We did not observe any changes in the GFAP cell area (*P* > 0.05, Fig. 3X).

**Fig. 4:**
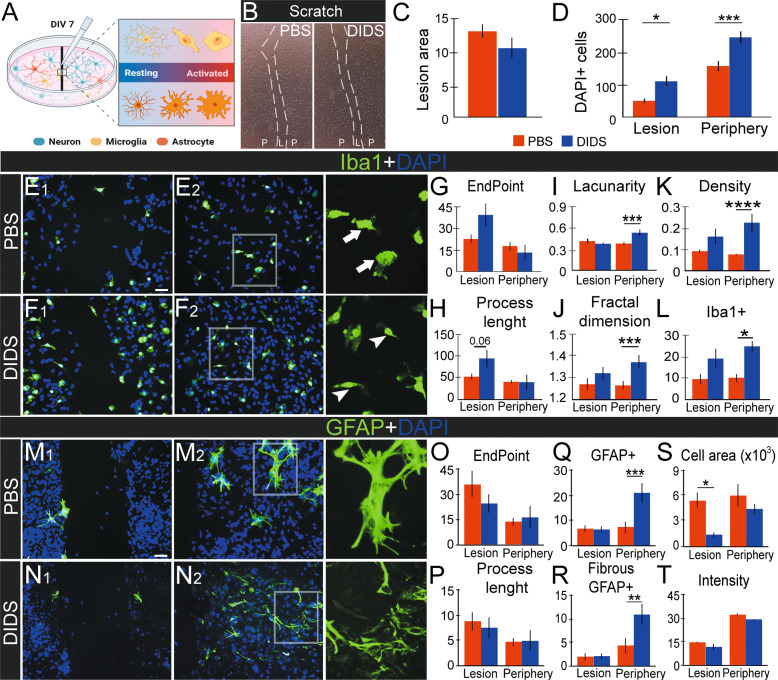
Effects of the VDAC1 inhibitor 4,4′-diisothiocyano-2,2′-stilbenedisulfonic acid (DIDS) on in vitro mechanical trauma model. **A** Schematic representation of mechanical scratch of the cell monolayer after seven days in vitro (DIV 7). **B** Live-cell imaging in PBS and DIDS treatment in the scratch experiment. The dotted line represents the limits of the scratched area. **C** Quantification of the scratch lesioned area treated with PBS (red) or DIDS [66] (*n* = 3). **D** Quantification of the number of 4–6-diamidino-2-phenylindole (DAPI) stained nuclei in PBS and DIDS treatment after scratching in the center of the lesion or its periphery (*n* = 8, and *p* < 0.05). **E**, **F** Representative image of Iba1 immunofluorescence (IF, green) counterstained with DAPI [66] with PBS or DIDS treatment in the center of the lesion (**E**_**1**_–**F**_**1**_) or its periphery (**E**_**2**_–**F**_**2**_) after scratch. The arrows indicate rod bipolar-shaped microglia and the arrowheads indicate ameboid cells. Quantification of microglial cell ramifications, showing the normalized number of endpoints per cell (**G**) and the mean of process length per cell (**H**). In both graphs, *n* = 8–9. Quantification of Iba1 heterogeneity considering microglia lacunarity (**I**, *n* = 12–25) and fractal dimension (**J**, *n* = 12–25). Quantification of parameters related to Iba1-positive cells, as density (**K**, *n* ≥ 12–25) and area (**L**, *n* ≥ 12–25). (M-N) Representative image of GFAP IF (green) counterstained with DAPI [66] with PBS or DIDS treatment in the center of the lesion (**M**_**1**_–**N**_**1**_) or its periphery (**M**_**2**_–**N**_**2**_) after scratch. Quantification of astrocyte ramifications, showing the normalized GFAP endpoint per cell (**O**, *n* = 8–11) and the mean of process length per cell (**P**, *n* ≥ 8–118). Representative graph of GFAP characteristics as the total number of GFAP cells (**Q**, *n* = 19–20), fibrous-like morphology GFAP cells (**R**, *n* = 19–20), GFAP cell area (**S**, *n* = 20–50), and GFAP labeling intensity (**T**, *n* = 8–11). *P < 0.05; **P < 0.01; ***P < 0.001; ****P < 0.0001 in one-way ANOVA followed by Sidak’s post-hoc. The illustrations were obtained using BioRender and Photoshop software.

We next aimed at the consequences of VDAC1 inhibition in the mechanical scratch model, treating the cells with PBS or 25 µM DIDS (Fig. 4A, B). We assessed the impacts of the treatment on cell viability by measuring MTT activity, and no differences were observed (data not shown). 25 µM DIDS did not alter the lesion area (*P* > 0.05, Fig. 4C), and a higher number of DAPI-nuclei was observed in the center (2.33 ± 0.99 vs. 4.87 ± 1.62, *P* < 0.05) and in the periphery of the lesion (158.11 ± 16.39 vs. 245.78 ± 18.24, *P* < 0.001, Fig. 4D). Iba1 labeling presented changes in heterogeneity and quantity with DIDS treatment, mostly in peripheral regions. Morphological changes were observed in the qualitative analyses since ‘bipolar/rod’ microglial shapes were seen with DIDS treatment (Fig. 4E, F). In the center of the lesion, Iba1+ cells tended to show more ramifications with DIDS treatment (9.44 ± 3.04 vs. 19.11 ± 4.64 Fig. 4G, H). DIDS triggered an increase in the number of Iba1+ cells (10.78 ± 2.01 vs. 27.78 ± 3.37, *P* < 0.05, Fig. 4L), microglia heterogeneity (lacunarity: 0.38 ± 0.02 vs. 0.53 ± 0.05, *P* < 0.001; fractal dimension: 1.26 ± 0.02 vs. 1.37 ± 0.03, *P* < 0.001, Fig. 4I, J), and cell density (0.08 ± 0.004 vs. 0.23 ± 0.04, *P* < 0.0001, Fig. 4K) in the periphery, but not in the center of the lesion.

Alterations were observed in the morphology of GFAP-positive cells (Fig. 4M, N), but not regarding astrocyte ramification (Fig. 4O, P, *P* > 0.05). We observed an increase in GFAP + cells (7.40 ± 2.15 vs. 21.00 ± 4.16, *P* < 0.05, Fig. 4Q) and GFAP + fibrous-like morphology (4.15 ± 1.62 vs. 10.75 ± 2.46, *P* < 0.05, Fig. 4R), both alterations observed in the periphery region. The area of GFAP + cells decreased in DIDS treatment in the center of the lesion (5,430 ± 1,026 vs. 1,384 ± 254, *P* < 0.05, Fig. 4S). The GFAP labeling intensity did not present any alteration in both central and periphery regions (*P* > 0.05, Fig. 4T).

Our in vitro data indicated that VDAC1 inhibition reduced cell death. DIDS treatment altered the microglial cell morphology and astrogliosis response, revealing its impact on glial cell recruitment and activation.

### VDAC1 is promptly regulated in the retina after the in vivo mechanical trauma

**Fig. 5:**
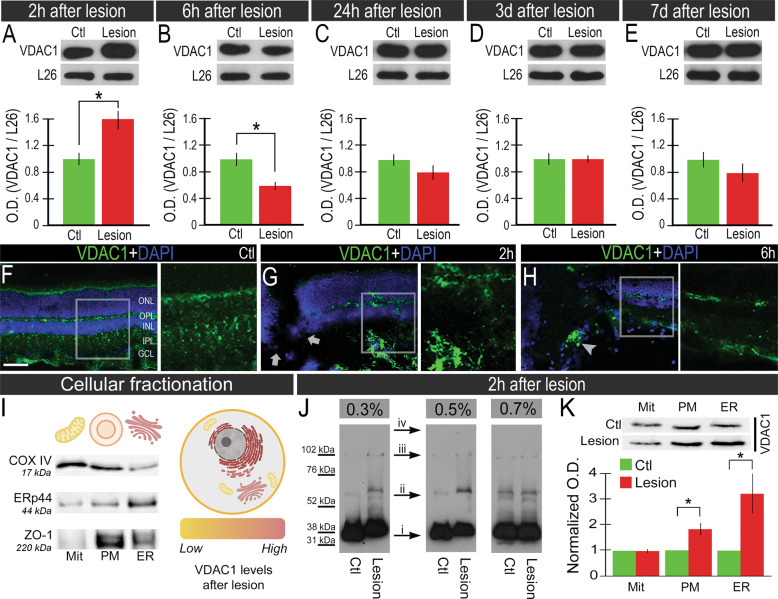
Voltage-dependent anion channel 1 (VDAC1) regulation after retinal mechanical injury. Western blot analysis of VDAC1 protein levels **A** 2 h, **B** 6 h, **C** 24 h, 3 days (**D**), and 7 days (**E**) after the lesion. Ribosomal L26 protein was used as an internal control. Representative VDAC1 immunofluorescence (IF, green) in transverse sections of **F** controls, **G** 2 h, and **H** 6 h after lesion, counterstained with 4′,6-diamidino-2-phenylindole (DAPI, blue). The arrows indicate the epicenter of the lesion, whereas head arrows indicate the accumulation of VDAC1 after the lesion. **I** Left: validation of cellular fractioning analyzing COX IV, ERp44, and ZO-1 proteins in mitochondria (Mit), plasma membrane (PM) and endoplasmic reticulum (ER). Right: representation of cell organelles according to VDAC1 levels after the mechanical trauma. **J** Crosslinking assay of VDAC1 2 h after lesion with 0.03%, 0.05% and 0.07% paraformaldehyde (PFA) concentrations, in which it was possible to identify monomers (i), dimers (ii), and other oligomers (iii and iv). **K** VDAC1 protein in mitochondria, plasma membrane, and endoplasmic reticulum in controls and 2 h after the lesion. Graph of normalized optical density (OD) of the subcellular fractionation experiment. Scale bar: 50 μm. All analyses considered *n* = 6 and *P < 0.05 in paired Student’s *t*-test. ONL outer nuclear layer, OPL outer plexiform layer, INL inner nuclear layer, IPL inner plexiform layer, GCL ganglion cell layer, The illustrations were created using BioRender software.

We next examined VDAC1 on in vivo trauma injury, which was rapidly and dynamically regulated. VDAC1 levels were 58 ± 13% higher (*P* < 0.05, Fig. 5A) and 40 ± 5% lower (*P* < 0.05, Fig. 5B) 2 h and 6 h after the lesion, respectively. VDAC1 levels were not altered 1, 3, and 7 days after lesion (*P* > 0.05 Fig. 5C–E). IF was employed to investigate VDAC1 distribution 2 h and 6 h after lesion. In the sham retina, VDAC1 accumulated in the inner segment of the photoreceptors, in both inner and OPLs, and GCL (Fig. 5F). At 2 h and 6 h after lesion, the distribution was similar, but with substantial variation in the signal intensity, which increased 2 h after lesion (Fig. 5G) and decreased 6 h after lesion (Fig. 5H).

We next aimed at the possible mechanisms by which the recruitment of VDAC1 might be involved in the neurodegenerative process. Two distinct experiments were performed, the cellular fractionation, and oligomerization assay. The cellular fractionation was validated by detecting COX IV for mitochondria and plasma membrane (UniProtKB - P19783), ERp44 for ER (UniProtKB - Q5VLR5), and ZO-1 for plasma membrane (UniProtKB - A0A0G2K2P5). Despite residual, faint bands in other fractions, stronger bands were detected according to the presumptive location of the proteins (Fig. 5I). Since the formation of VDAC1 oligomers is an important event to induce cell death, the oligomerization was verified in the crosslinking assay. Using three concentrations of paraformaldehyde (PFA), it was possible to identify bands in molecular weights corresponding to VDAC1 dimers, trimers, and multimers (Fig. 5J). Moreover, we detected VDAC1 in mitochondria, ER, and plasma membrane (Fig. 5K). VDAC1 levels increased in the plasma membrane (82 ± 22%, *P* < 0.05) and ER (223 ± 23%, *P* < 0.05). 2 h after the lesion.

### Pharmacological inhibition of VDAC1 protects the retina after mechanical trauma

**Fig. 6:**
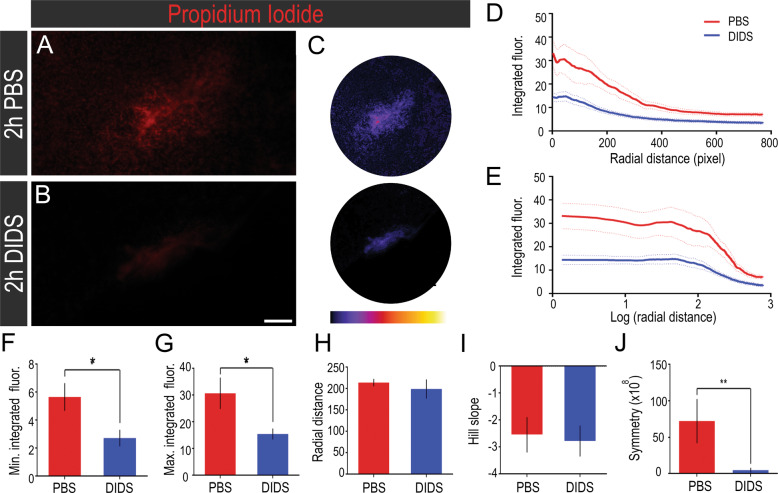
In vivo pharmacological inhibition of voltage-dependent anion channel 1 (VDAC1) using 4,4′-diisothiocyano-2,2′-stilbenedisulfonic acid (DIDS) attenuates propidium iodide (PI) spread in the regions next to the lesion. PI staining in (**A**) PBS- and (**B**) DIDS-treated retinas 2 h after lesion. **C** Representative image of the analyzed region in PBS- and DIDS-treated retinas and the respective scale showing lower (black/blue) to higher (yellow/white) signal intensity, as generated by ImageJ software. **D** Integrated signal intensity as a function of radial distance from the lesion. (**E**) Integrated signal intensity as a function of the logarithm of radial distance from the lesion. **F** Minimal signal intensity after treatment obtained from the five-parameter logistic (5PL) curve fit (*n* = 6–10). **G** Maximal signal intensity after treatment obtained from 5PL curve fit (*n* = 6–10). **H** Hill slope obtained from 5PL curve fit (*n* = 6–10). **I** Distance from the focus of the lesion in which the integrated signal intensity corresponds to an inflection point obtained from 5PL curve fit (*n* = 6–10). **J** Symmetry was obtained from 5PL curve fit (*n* = 6–10). *P < 0.05; **P < 0.01 in Student’s *T*-test. Scale bar: 100 μm.

We next aimed to examine the role of VDAC1 in retinal degeneration by inhibiting its activity. Propidium iodide (PI) was used to stain dying cells in retinal explants after PBS (Fig. 6A) or DIDS (Fig. 6B) treatment. A high PI intensity labeling was observed near the lesion focus, with a monotonic decrease with the distance (Fig. 6C, D). The experimental data points fitted an asymmetric logistic function, which generated a five-parameter logistic (5PL) curve (Fig. 6E). A decreased PI staining was observed in DIDS-treated retinas, as revealed by the minimum (5.66 ± 0.99 vs. 2.73 ± 0.60, *P* < 0.05, Fig. 6F) and maximum (30.74 ± 5.84 vs. 15.50 ± 2.08, *P* < 0.05, Fig. 6G) integrated fluorescence values. The inflection point in the curve did not change (Fig. 6H), nor the slope (Fig. 6I), whereas the symmetry coefficient decreased with DIDS treatment (72.3 × 10^8^ ± 30.3 × 10^8^ vs. 4.79 × 10^8^ ± 3.25 × 10^8^, *P* < 0.01, Fig. 6J).

### Pharmacological inhibition of VDAC1 attenuates both cell death and microglial activation, disrupting neuroinflammatory response

**Fig. 7:**
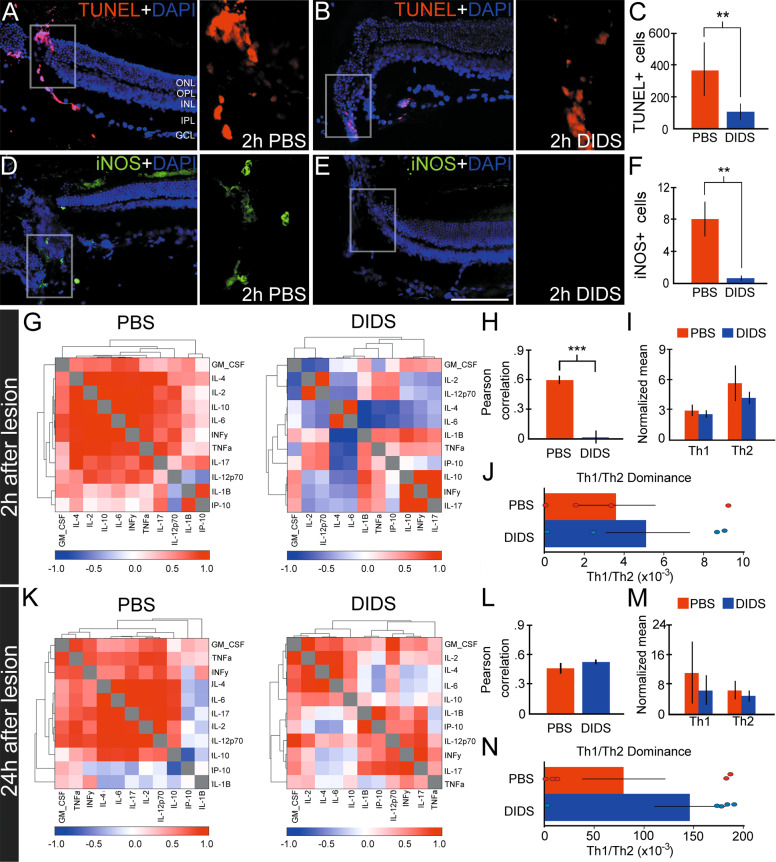
Impact of pharmacological inhibition of voltage-dependent anion channel 1 (VDAC1) using 4,4′-diisothiocyano-2,2′-stilbenedisulfonic acid (DIDS) on cell death, inducible nitric oxide synthase (iNOS) distribution and neuroinflammatory response after retinal lesion. TUNEL labeling in **A** PBS- and **B** DIDS-treated retinas two hours after lesion. **C** Quantification of TUNEL-positive cells in the lesion’s focus (*n* = 7). iNOS immunofluorescence in (**D**) PBS- and (**E**) DIDS-treated retinas 2 h after lesion. (**F**) Quantification of iNOS-positive cells in the epicenter of the lesion (*n* = 7). ONL outer nuclear layer, OPL outer plexiform layer, INL inner nuclear layer, IPL inner plexiform layer, GCL ganglion cell layer. Scale bar: 100 μm. **G** Cytokines heatmap of PBS- and DIDS-treated retinas 2 h after the lesion. Pearson’s correlation values range from −1.0 [66] to 1.0 (red). **H** Mean of Pearson’s correlation analysis in PBS- (red) and DIDS- [66] treated retinas 2 h after lesion. **I** Graph of the normalized mean of Helper T cell type 1 and 2 cytokines (Th1 and Th2) 2 h after the lesion. Th1-type cytokines tend to produce perpetuating pro-inflammatory responses, whereas Th2-type cytokines that include interleukins 4, 5, 10, and 13 are associated with promoting IgE- and eosinophilic-related processes, and thereby a balance with an anti-inflammatory bias. **J** Th1/Th2 dominance graph in PBS- and DIDS-treated retinas 2 h after the lesion. All cytokine analyses in 2 h were performed using *n* = 4. **K** Heatmap of cytokines of PBS- and DIDS-treated retinas 24 h after the lesion. **L** Mean of Pearson’s correlation analysis in PBS- and DIDS-treated retinas 24 h after the lesion. **M** Graph of the normalized mean of Th1 and Th2 cytokines 24 h after the lesion. **N** Th1/Th2 dominance graph in PBS- and DIDS-treated retinas 24 h after the lesion. All cytokine analyses in 24 h were performed using *n* = 5. **P < 0.01, ***P < 0.001. All analyses were performed using Students’ *t*-test.

We also evaluated the distribution and number of dying cells using TUNEL in PBS- (Fig. 7A) and DIDS-treated (Fig. 7B) retinas. The number of TUNEL-positive cells decreased with DIDS (369.57 ± 60.35 vs. 108.14 ± 21.05, *P* < 0.01, paired *t*-test, Fig. 7C). We next investigated whether DIDS treatment could attenuate inflammation after lesion. Nitric oxide synthase (iNOS)-positive cells were observed 2 h after lesion (Fig. 7D). DIDS prevented the recruitment of these cells (Fig. 7E), which were rarely seen in the lesion (8.00 ± 2.18 vs. 0.57 ± 0.30, paired t-test, *P* < 0.01, Fig. 7F).

Considering the role of microglial cells in neuroinflammatory response, we examined cytokines using multiplex bead assay. A high correlation in analytes was observed 2 h after the lesion, revealing coordination in the inflammatory response. This correlation significantly decreased with DIDS treatment (0.60 ± 0.05 vs. 0.01 ± 0.08, *P* < 0.001, unpaired t-test, Fig. 7G, H), even without changes in the normalized mean and ratio of Th1/Th2 (Fig. 7I, J), a measure of cytokine dominance established by IFNy/IL-4 ratio. DIDS treatment did not affect correlation (Fig. 7K, L), nor Th1/Th2 normalized mean and dominance (Fig. 7M, N) 24 h after the lesion.

## Discussion

The findings in this study are summarized as follow: (i) downregulation of VDAC1 levels triggers degeneration in the retina in an independent-caspase process that simultaneously regulates SOD1; (ii) mechanical trauma in the retina recruits VDAC1 and SOD1, and induces oxidative stress and secondary cell death; (iii) inhibition of VDAC1 decreases cell death on in vitro model of oxidative stress, and this effect depends on the presence of microglial cells and astrocytes; (iv) VDAC1 pharmacological inhibition decreases the secondary cells loss on in vitro mechanical trauma, impacting the polarization of microglial cells and astrocytes; (v) mechanical trauma of the retina promptly regulates VDAC1, which was shown to form multimers, and translocates to the plasma membrane and ER; (vi) in vivo inhibition of VDAC1 decreases microglial cells activation and promotes neuroprotection by reorganizing the inflammatory response.

VDAC1 knockdown induced inward folds of the ONL associated with local retinal detachments and thinning of the INL. This pattern resembles rat retina aging [34, 35], known as the rosette. Rosette formation is associated with changes in mitochondrial morphology [36], which causes dysregulation of mitochondrial processes [37]. We observed that VDAC1 expression is rapidly regulated after retinal mechanical trauma. Regulation of VDAC1 could be associated with general processes, encompassing restoring metabolic pathways and mitochondrial adaptation. Our data revealed that VDAC1 is upregulated in the ER after trauma injury, where Ca^2+^ stores can be mobilized, triggering neurodegenerative signaling [17]. Similarly, VDAC1 assembling and upregulation in the ER may play an essential role in neurodegenerative disorders [38]. To study the VDAC1 in the mechanical lesion, we used DIDS, which reversely binds to VDAC1 and blocks its action [29–31].

DIDS treatment decreased cell death spreading after lesion, which is in accordance with previous reports on in vitro ischemia model [39] and spinal cord injury [40]. Cell viability according to the distance from the lesion fitted a sigmoidal asymmetric curve. Considering the upper asymptote as the focus of the lesion and the lower as the less affected surrounding areas, the transition can be viewed as the penumbral region, in which secondary cell death occurs. The impact of DIDS on the penumbra region was confirmed with TUNEL labeling, since blocking of VDAC1 decreased cell death.

Considering the fast upregulation of VDAC1 after retinal lesion and that the protein levels determine its equilibrium between multimers [41], we investigated VDAC1 oligomerization. We could not find structures superior to trimers, while the oligomers associated with cell death induction are hexamers [41, 42]. We focused on pathways in which VDAC1 could be involved in cell death. Independent groups found VDAC1 in the plasma membrane [18–20], synaptosome [43], and ER [21]. In the ER, VDAC1 is essential for integrating this organelle and mitochondria [21, 44], regulating Ca^2+^ signaling and ionic balance [17, 23]. This porin is also present in the plasma membrane, has enzymatic activity in healthy neurons, and acts as a channel in apoptotic neurons [16]. Once in other locations, such as ER and plasma membrane, VDAC1 can be associated with Bcl2 proteins, increasing Ca^2+^ levels and triggering apoptosis [45].

VDAC1 allows ATP release, which can activate microglia [46] and attract phagocytic cells [47, 48]. After activation, microglia can acquire the M1 or M2 phenotype. While M1 delivers pro-inflammatory cytokines and mediators, M2 releases anti-inflammatory factors [49]. Our in vitro experiments showed that DIDS resulted in a higher microglia density and a tendency of hyperbranched morphology in oxidative stress induction and mechanical scratch degeneration models. Astrocytes were seen more ramified in the presence of DIDS, indicating that VDAC1 may be involved in the glial scar formation after mechanical trauma. Notably, DIDS treatment rearranged the immune response in vivo, decreasing anti- and pro-inflammatory molecules, revealing that VDAC1 participates in the immunoregulatory system balance. Accordingly, the employment of VDAC1-interacting molecules, such as VBIT-12, indicated the involvement of VDAC1 in the inflammatory response [50].

In conclusion, our data show that VDAC1 integrates signals to determine cell survival. Reduced levels affect energy-demanding processes, which explains why VDAC1 ablation has severe consequences for neurons. In excess, this protein can induce cell death since translocation to the ER and plasma membrane seems a particular hallmark of its lethal effects, possibly constituting a common pathway in several diseases. Altogether, these data indicate VDAC1 as a potent therapeutic target for traumatic and chronic neurodegenerative disorders.

## Material and methods

### Ethics statement

These experiments were conducted under the guidelines of the NIH and the Brazilian Scientific Society for Laboratory Animals. The experimental protocol was approved by the Ethics Committee in Animal Experimentation of Federal University of ABC (CEUA-UFABC, protocol number 17/2015 and 1102011018).

### Retinal primary cell culture

P0-P4 animals were euthanized, retinas were dissected and washed with DMEM/F12 medium (Gibco, #11320033) before being chemically digested with papain solution (Gibco, #88285) for 30 min at 37 °C. The papain digest solution was replaced with DMEM. The following steps were performed as previously described [51]. After precipitated homogenization with Neurobasal-A medium supplemented with 10% Fetal Bovine Serum (FBS, LCGBio, Brasil, #10-BIO500), 1% l-Glutamine (Gibco, #25030149) and 2% B27™ Supplement (Gibco, # 17504044), the solution containing the retinal cells was distributed to the wells previously treated with Poly-D-lysine (Gibco # A3890401). Dissociated cells were counted and plated (3 × 10^4^/well in 96 well-plates and 18 × 10^4^/well in 24 well plates), and the cell culture medium was renewed every 48 h. B-27™ Supplement was used in all experiments, except those carried out with H_2_O_2_, in which B-27™ Supplement (50X, Gibco, #10889038) without antioxidants was employed.

### MTT tetrazolium reduction assay

The cellular viability was determined by the reduction test of 1-(4,5-dimetiltiazol-2-il)-3,5-difenilformazan (MTT). For H_2_O_2_ oxidative stress model, the cells were pre-incubated with VDAC1 inhibitor, 4,4′-diisothiocyano-2,2′-stilbenedisulfonic acid (DIDS; Sigma Aldrich, cat# D3514,) 30 min before H_2_O_2_ incubation. For mechanical injury, DIDS was included right after the scratch of the cell monolayer. All treatments were performed for 24 h in 96 wells plate and 5% CO_2_. After the incubation period, a fresh medium replaced the cell medium, and 10 μL of MTT solution (5 mg/ml) were added. After 2 h of incubation, the cell medium with MTT solution was replaced by pure DMSO (Synth, Brasil, #D1011.01.BJ) without dilution. The absorbance values were obtained in an ELISA plate reader (SpectraMax M5) at 570 nm (reference at 620 nm). Data were normalized by values obtained from the untreated wells, and experiments were performed at least three independent times.

### Oxidative stress induction

Oxidative stress was induced in primary cell culture with hydrogen peroxide (H_2_O_2_, Neon Comercial Ltda #01984). H_2_O_2_ solution was quantified using spectrophotometry to obtain the final concentrations ranging from 0.5 µM to 100 µM. After 7 days in vitro (DIV), primary cell cultures were treated with the H_2_O_2_ solution for 24 h. For VDAC1 inhibition, cell cultures were pre-treated with 4,4′-diisothiocyano-2,2′-stilbenedisulfonic acid (DIDS; Sigma Aldrich, cat# D3514, at 25 or 50 µg/mL concentrations) or the vehicle (PBS) during 30 min before H_2_O_2_ exposition.

### Mechanical scratch of the cell monolayer

After 7 DIV, a scratch injury was adapted from the protocol described by Liang [52]. With a pipet tip, the bottom of the well (15.22 mm) was scraped from top to bottom in a straight line. Immediately after the scratch procedure, the cell medium was withdrawn to remove the debris, and the medium was supplemented with 25 µM DIDS or the vehicle (PBS). After 24 h, cells were fixed with 4% PFA. The images were obtained using Evos XLCore Imaging System (ThermoScientific, #AMEX1000) right after the scratch procedure and 24 h later. The scratched area was calculated using ImageJ software (version 1.50i, National Institute of Health, Bethesda, MD, USA), and the comparisons were performed in one-way ANOVA followed by Sidak post-hoc test.

### Animal procedures

Experiments were carried out with male and female Long Evans rats (*Rattus norvegicus*) ranging from 60 to 90 postnatal days. Since animals were randomly chosen to form experimental groups, the identification was not blinded. For VDAC1 *knockdown*, morpholino antisense oligonucleotides (MO, GeneTools, Philomath, USA) were designed to target VDAC1 mRNA (5′-ATATGTGGGAGGCACAGCCATGTTC-3′) specifically. As a control, a scramble MO was used (5′-CCTCTTACCTCAGTTACAATTTATA-3′). Both oligonucleotides were diluted to 0.5 mM in PBS. Animals were anesthetized under isoflurane and the eye was pulled apart with curved dressing forceps to facilitate eye manipulation. Using a Hamilton syringe, 5 µL of MO (0.5 mM) were injected in the subretinal space. Then, the animals were euthanized after 5 days, and the retinas were carefully dissected.

The procedures for the mechanical trauma experiments were previously described [53]. Briefly, animals were anesthetized by intraperitoneal injection of a mixture of ketamine (75 mg/kg) and xylazine (10 mg/kg) and submitted to six local mechanical lesions in the retina, performed using a thin needle (28-gauge, 12.7 mm). After different periods (2 h to 7 d), animals were euthanized with a lethal dose of urethane (1 g/kg), and the retinas were removed and dissected for the different assays. For in vivo VDAC1 inhibition, DIDS was diluted in PBS. Using a Hamilton syringe, intraocular injection of 7 μL of DIDS (1 mM) or PBS was performed. After 2 h, the retinas were carefully dissected for different analyses.

### Hematoxylin and eosin staining

Fixed retinas were cryosectioned and rehydrated with xylene and serial decreasing ethanol concentrations (100%, 95%, 80%, 5 min each). After washing with deionized water (7 min), the slides were stained with Mayer’s hematoxylin solution (1.0 g hematoxylin, 1000 ml distilled water, 0.2 g sodium iodate, 50 g potassium alum, 50 g chloral hydrate, 1.0 g citric acid) for 3 min and washed with water for additional 3 min. Then, sections were stained with eosin solution (1.0 g eosin Y, 80 ml distilled water, 320 ml 95% ethanol, 0.4 ml glacial acetic acid) for 7 min and posterior dehydration and washing xylol. The slices were dried and closed. The retinal sections were analyzed under a bright-field microscope (DM 5500, Leica Microsystems, Germany) coupled to a camera for image capture (DFC 365 FX, Leica Microsystems, Germany). The area of specific retinal layers was measured using ImageJ software.

### Western blotting

Retinas were homogenized in RIPA buffer (50 mM Tris, 150 mM NaCl, 0.1% SDS, 0.5% sodium deoxycholate, 1% Triton X-100, and protease inhibitors). Homogenates were centrifuged for 20 min at 14.000 G, 4 °C to remove insoluble material. Protein concentration was determined by the BCA method (Thermo Scientific, Rockford, IL, USA, catalog # 23225) and bovine serum albumin was used as the standard, following manufacturer protocol. Total protein was separated in a 10% polyacrylamide electrophoresis gel and transferred to nitrocellulose membranes. Blots were blocked with 5% non-fat milk in the TBST buffer for 2 h at room temperature. After rinsing with TBST, blots were incubated overnight with primary antibodies in TBST/3% non-fat milk, according to Table 1. After incubating with primary antibodies, blots were rinsed with TBST and incubated with goat anti-peroxidase (ECL^TM^ kit; Amersham, Buckinghamshire, England) for 2 h at room temperature. Detection of labeled proteins was achieved by using the enhanced chemiluminescent system (ECL^TM^ kit; Amersham). Measurements of optical densities (O.D.) of the bands were performed using ImageJ software (National Institute of Mental Health, Bethesda, Maryland, USA). O.D. values were normalized using the value obtained in the control group. L26 protein or amido black total protein staining were used as intern control, according to the indication in the respective figure legend. The SOD1/VDAC1 O.D. correlation was calculated using Pearson’s coefficient.

**Table 1:** Antibodies used in this study.

Protein	Supplier	Cat#	WB	IF in vivo	IF in vitro
Brn3a	Santa Cruz	sc-8429	1:1000	1:100	---
Calbindin	Sigma	C9848	1:1000	1:100	---
Cleaved caspase-3	Millipore	04–439	1:1000	---	---
COX IV (3E11)	Cell Signaling	4850	1:1000	---	---
ERp44	Cell Signaling	3798	1:1000	---	---
GFAP	Sigma	G3893	---	---	1:500
Iba1	Abcam	ab5076	---	---	1:500
iNOS	Millipore	06–573	---	1:100	---
L26	Abcam	ab59567	1:1000	---	---
PKCα	BD Transduction	612698	1:1000	1:100	---
SOD1	Sigma	SAB2500976	1:1000	1:75	---
VDAC1	Abcam	ab135585	1:1000	1:400	---
ZO-1	Invitrogen	617300	1:1000	---	---

### Crosslinking assay

This method was adapted from [54]. Two hours after lesion, the retinas were dissected and incubated in three PFA concentrations (0.03%, 0.05%, or 0.07%) for 10 min at room temperature, being posteriorly washed twice in 1.25 M glycine for 5 min at 4 °C. After, retinas were homogenized in the RIPA buffer and prepared for western blot following the previously described procedures without reducing agents.

### Cellular fractionation

This method was adapted from [55, 56]. Two hours after the lesion, retinas were dissected and homogenized in H-buffer (0.32 M sucrose, 4.0 mM HEPES, and protease inhibitors, pH 7.4). The homogenates were centrifuged 740 G for 5 min at 4 °C to pellet nuclei. The first resulting supernatant was centrifuged at 9000 G for 10 min at 4 °C to pellet mitochondria. The second resulting supernatant was centrifuged 20,000 × *g* for 30 min at 4 °C to pellet plasma membrane. The supernatant was collected in a new tube to analyze the ER and cytosol. All the pellet fractions were resuspended in the RIPA buffer. Then, the proteins were quantified and prepared for protein detection as described above. The method was validated using antibodies raised against the proteins COX IV, ERp44, and ZO-1 [57, 58], as described in Table 1.

### Propidium iodide uptake assay

Retinas were dissected and incubated in the PI solution (20 mg/mL, diluted in PBS) for 2 h at 4 °C. Next, retinas were washed with PBS and mounted on slides for microscopy analyses. The images were analyzed with the ImageJ software. Images were acquired at 10× magnification, maintaining the epicenter of the lesion in the middle of the microscopy field. On the analysis software, it was selected a squared region around the lesion, and it was used a radial quantification plugin (http://rsb.info.nih.gov/ij/plugins/radial-profile.html), determining the intensity of the signal on the concentric circumferences. Student’s *t*-test was performed as the statistical analysis.

### Immunofluorescence

The IF assay was performed for in vitro and in vivo experiments. For in vivo experiments, eyes were dissected out and fixed for 8 h in 4% PFA in phosphate buffer 0.1 M pH 7.3 and cryoprotected in 30% sucrose solution for at least 24 h at 4 °C, embedding using O.C.T. compound (Sakura Finetek, Torrance, CA, USA), and retinas were sliced transversely (12 μm) using a cryostat (Leica CM1860 UV, # 76404-216). For in vitro experiments, the cells were fixed in PFA 4% for 1 h at 4 °C under constant agitation, and the non-specific binding sites were removed by two washes using PBS 0.9% and PBS 0.9% + 0.4% Triton X-100 and PBS 0.9% + 0.1% Tween 20. Retinal cells and sections were incubated overnight with primary antibodies in a solution containing 5% normal donkey serum and 0.3% Triton X-100 in PBS at 4 °C over agitation (for cells) or room temperature (for tissue). All antibodies and specific concentrations used in this study are listed in Table 1. On the following day, the secondary antibody was added to the cells and tissues, composed by donkey antiserum against rabbit, mouse, or goat IgG tagged to Alexa 488 (1:500–1:3000, Invitrogen) diluted in 3% normal donkey serum containing 0.5% Triton X-100 in PBS for 2 h (for tissue) and 3 h (for cells) at room temperature. For double-labeling experiments, we used secondary antibodies conjugated to Alexa 546 and Alexa 647 (1:500–1:3000, Invitrogen). Experimental controls were prepared by omitting the primary antibodies. Nuclei were counterstained using 4′,6-diamidino-2-phenylindole (DAPI, 1:5000) by incubating sections at room temperature for 10 min. For in vivo experiments, sections were mounted using VectaShield (Vector Labs, Burlingame, CA, USA). All micrographs were analyzed in a Nikon TS100F inverted microscope (Nikon Instruments Inc., Melville, NY, USA). Colocalization analyses were performed using Manders’ coefficient in Coloc2 ImageJ plugin. The pixel profile was obtained on NISelements AR (Nikon Instruments Inc., Melville, NY, USA).

For in vitro analyses, the microglial branching and morphology parameters were quantified as previously reported [59]. Similar analyses were performed for the GFAP marker. For cell counting, six different regions of samples from three animals were used. The positive cells were counted blind-manually using ImageJ Cell Counter (available at https://imagej.nih.gov/ij/plugins/cell-counter.html), and the number of cells was normalized by total cells DAPI+ in the same region. All images analyzed were acquired with the same exposition intensity.

Figures were prepared using Adobe Photoshop CC 2014 (Adobe Systems Inc., San Jose, CA, USA). Manipulation of the images was restricted to brightness and contrast adjustments of the whole image.

### Terminal deoxynucleotidyl transferase (TdT)-mediated dUTP nick-end labeling (TUNEL) assay

The TUNEL assay was performed as previously described [2] Briefly, in vivo mechanical trauma retinas treated with DIDS or vehicle was collected after 2 h, fixed in PFA 4% 30 min with picric acid and cut transversely (12 μm) using a cryostat (Leica CM1860 UV, #76404-216). The TUNEL labeling was accomplished by incubation in TdT buffer for 10 min at room temperature followed by incubation with a biotinylated 2-deoxy-uridine-5-triphosphate (dUTP) (Roche Molecular Biochemicals, Mannheim, Germany). The reaction was terminated using the stop reaction buffer and washed in PBS. The sections were mounted using the ProLong Antifade kit (Molecular Probes). Positive cells were counted with TUNEL Cell Counter [60].

### Protein oxidation measurements

Retinal samples were exposed to RIPA solution (RadioImmuno Precipitation Assay Buffer = 150 mM NaCl, 5 mM EDTA, 1 mM dithiothreitol, 1% Triton X-100, 0.5 mM sodium dioxycholate, and 0.1% SDS in 50 mM Tris at pH 7.5) in which they were homogenized and lysed by vortexing for 15 seconds, then placed in an ice bath for 30 min and centrifuged at 14,000 × *g* for 20 min at 4 °C [61]. An aliquot of the supernatant was removed to quantify proteins according to the Lowry method. The supernatant was treated as described by Levine and Postu [62, 63]. In aliquots containing up to 1 mg of protein were added 500 µL of 2,4-dinitrophenylhydrazine in 2 M HCl solution and left to rest for 1 h. Then 500 µL of trichloroacetic acid (TCA) (20%) was added and centrifuged at 11,000 × *g* for 3 min, the supernatant was discarded, and the pellet was washed three times with ethanol: ethyl acetate (1:1) solution. The supernatant was discarded and the pellet was dissolved in 600 µL of 6.0 M guanidine solution in 20 mM potassium phosphate buffer (pH 2.3, adjusted with trifluoroacetic acid), and the carbonyl was measured in a spectrophotometer at a wavelength of 370 nm. Carbonyl quantification was calculated by the value of molar extinction coefficient (*ε*) which is equal to 22,000/M cm. After reading, new protein quantification was performed using the Bradford method, and the results were expressed as nmol of carbonyl/mg of protein. Measurements were done in triplicate (*n* = 5).

### Lipid oxidation measurements

Retinal samples were frozen, washed with a 0.9% NaCl solution, then homogenized in a 50 mM phosphate buffer solution, pH 7.4 containing 1.15% KCl, and centrifuged at 960 *x* g for 15 min. After that, an aliquot corresponding to 200 µl was removed and then added a solution containing 20% (v/v) of trichloroacetic acid (TCA) and 0.8% (v/v) of thiobarbituric acid and heated to 95 °C for 25 min. After the reaction time, the samples were centrifuged at 300 × *g* for 5 min, and the product was measured in a spectrophotometer at a wavelength of 535 nm, and malonaldehyde (MDA) was determined according to the value of *ε* 1.49 × 10^5^/ M.cm. Protein quantification was performed using the Lowry method and the values expressed in nmol of MDA per mg of protein [61, 64]. Measurements were done in triplicate (*n* = 5).

### Multiplex magnetic bead array

Vehicle- and DIDS-treated retinas were harvested 2 and 24 h after the mechanical lesion and homogenized in an EDTA-free Protease Inhibitor Cocktail (Roche, #11836170001). Homogenates were centrifuged for 20 min at 14,000 G at 4 °C. BCA method was used to determine protein concentration, and bovine serum albumin was used as the standard. The following steps were performed according to the supplier’s instruction using the rat cytokine/chemokine magnetic bead panel (Millipore, #RECYTMAG-65K), and the cytokines GM-CSF, IL-4, IL-1B, IL-2, IL-6, IL-10, IL-12p70, INFy, IL-17, IP-10, and TNFa were simultaneously quantified. Cytokines values were normalized by the total protein concentration of each sample. Th1 and Th2 cytokines were divided according to the literature. Th1/Th2 dominance was determined by the INFy/IL-4 ratio. The mean of Pearson’s correlation was obtained in the correlation matrix report of heatmap statistical analysis using NCSS software (NCSS, LLC. Kaysville, Utah, USA, ncss.com/software/ncss.).

### Statistical analysis

Data are expressed as the mean ± standard error of the mean of at least three independent experiments for in vitro and at least five for in vivo analysis. The estimation of the sample size of each experiment, including animal experiments, was determined based on the literature and experimental *n* reported in the respective figure legend. The variance of each data group was determined and compared to employ the proper statistical test. All data meet the tests’ assumptions, for instance, normal distribution. Outliers were determined by multiplying the interquartile range (IQR) by 1.5, and the value was subtracted or added to the first or third quartile, respectively.

All comparisons were performed according to the figure legends. A *p* value < 0.05 was considered statistically significant. The statistical analysis was performed using GraphPad Prism version 6.00 for Windows (GraphPad Software, La Jolla, California, USA).

## Supplementary information

AJ Checklist

Supplemental material

## Data Availability

Data sharing is not applicable to this article as no datasets were generated or analyzed during the current study.
