# Supplementary material for: VDAC1 regulates neuronal cell loss after retinal trauma injury by a mitochondria-independent pathway
Source: Cell Death Dis. 2022 Apr 21;13(4):393. doi: 10.1038/s41419-022-04755-3 (PMC9023530; doi:10.1038/s41419-022-04755-3)

Supplementary Material

*This file is a supplementary material containing the original immunofluorescence micrographs and western blot data described in* VDAC1 regulates neuronal cell loss after retinal trauma injury by a mitochondria-independent pathway *manuscript.*

The original Western blot figures are described in Figure 1. The ladder defines the approximately molecular weight in the left lane. The weight is in kDa. The black arrow indicates the protein bands. “+” indicates scramble MO condition, and “-“ indicates the VDAC1 MO intervention.

Figure 1:


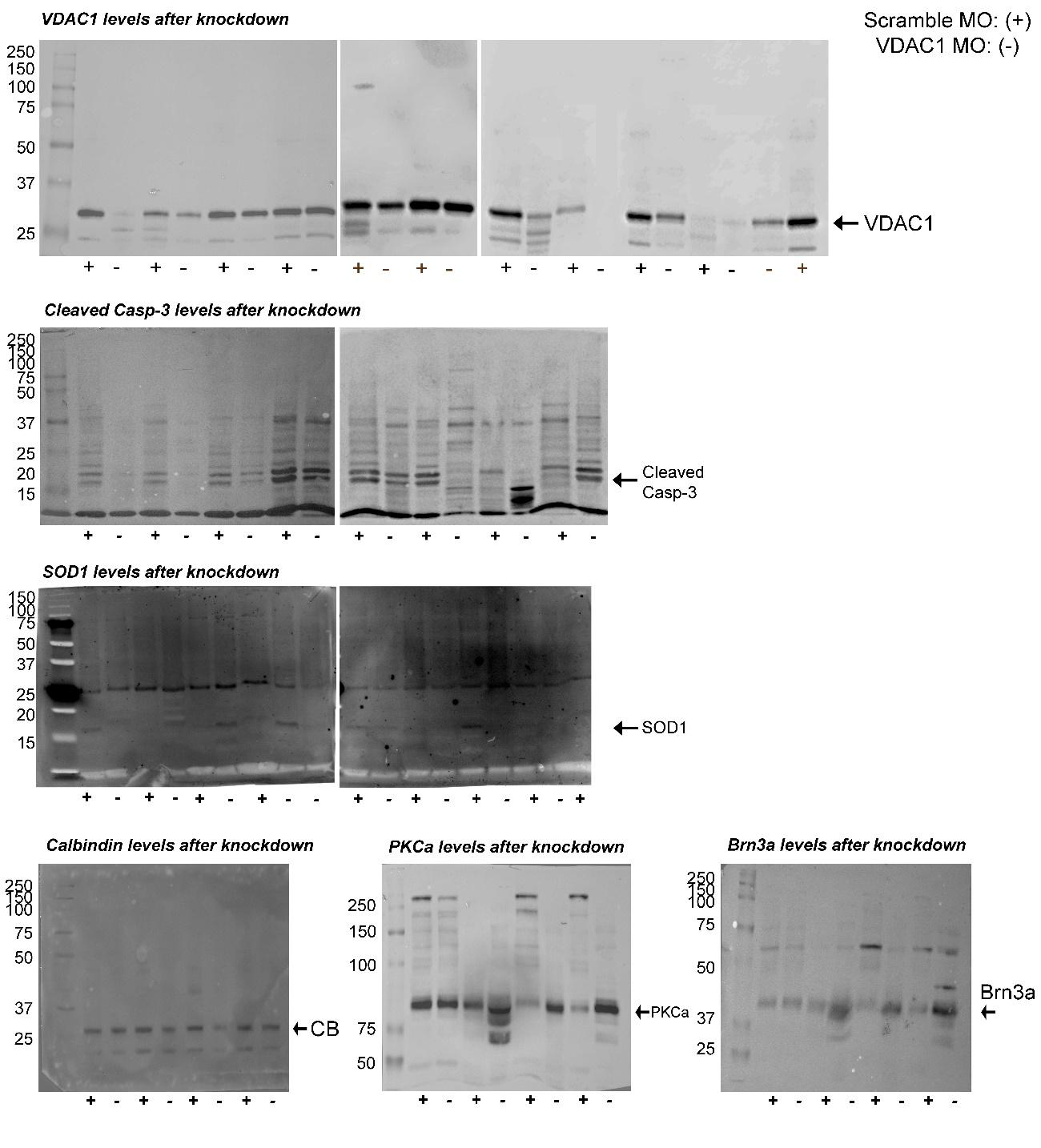


Figure 2 contains the original digitalized blots obtained after MO intervention. The images were not submitted to digital edition.

Figure 2:


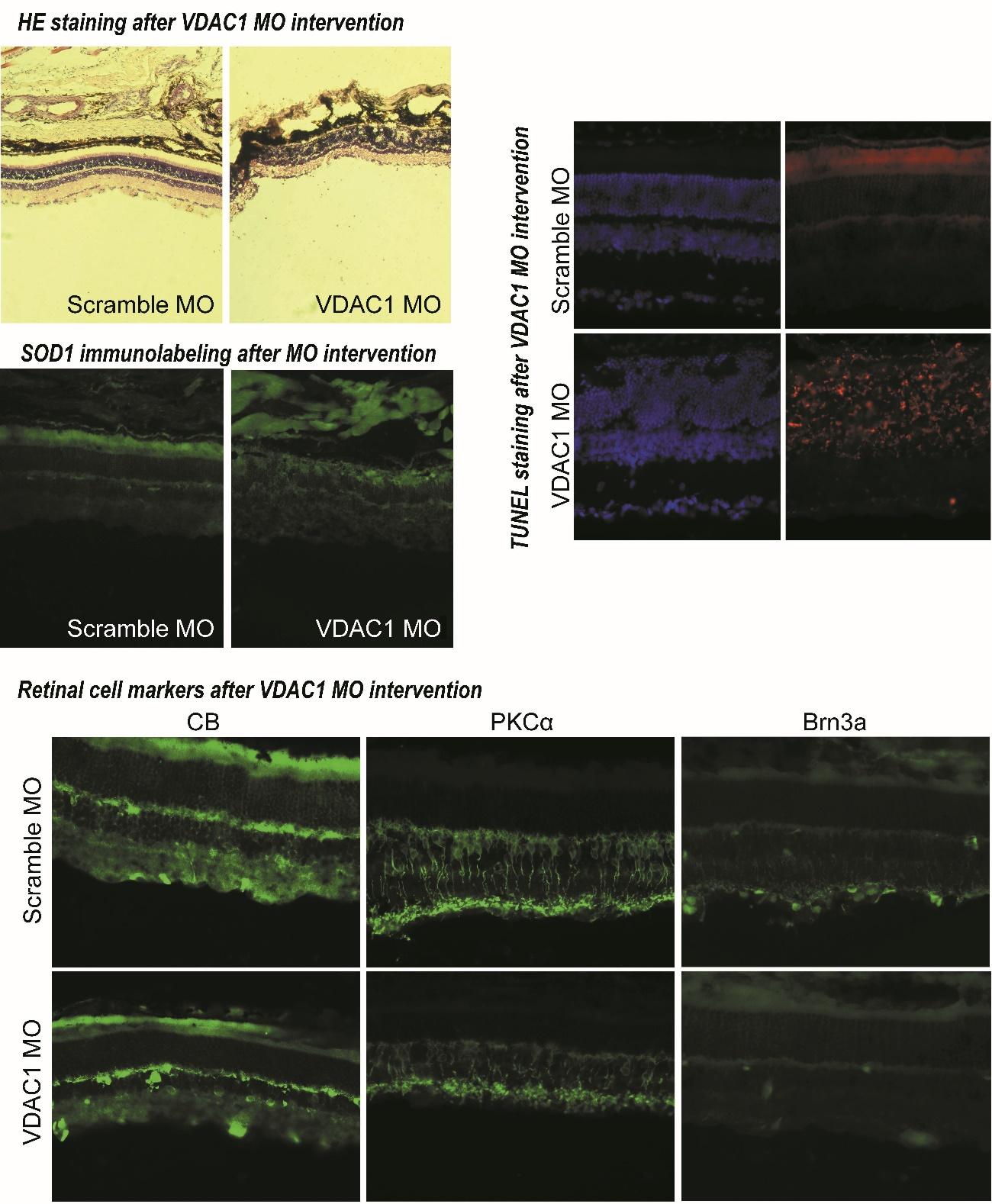


Figure 3 shows the original figures obtained after *in vitro* chemical and physical models of degeneration treated with DIDS inhibitor. Moreover, EVOS bright field original images were shown.

Figure 3


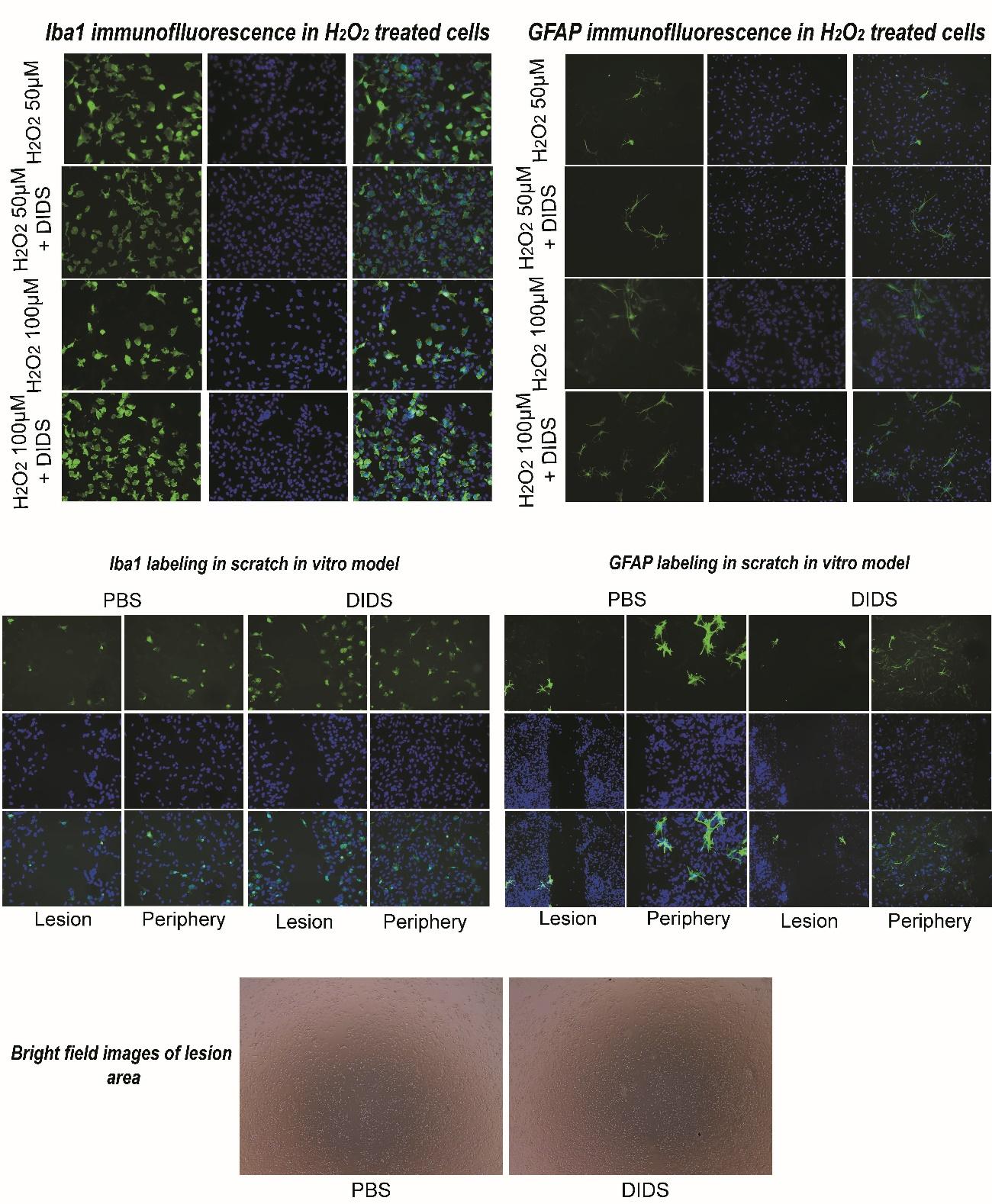


Figure 4 compilates the mechanical *in vivo* retinal trauma and the consequences of specific markers in the cell death progression, or after DIDS intraocular treatment.

Figure 4:


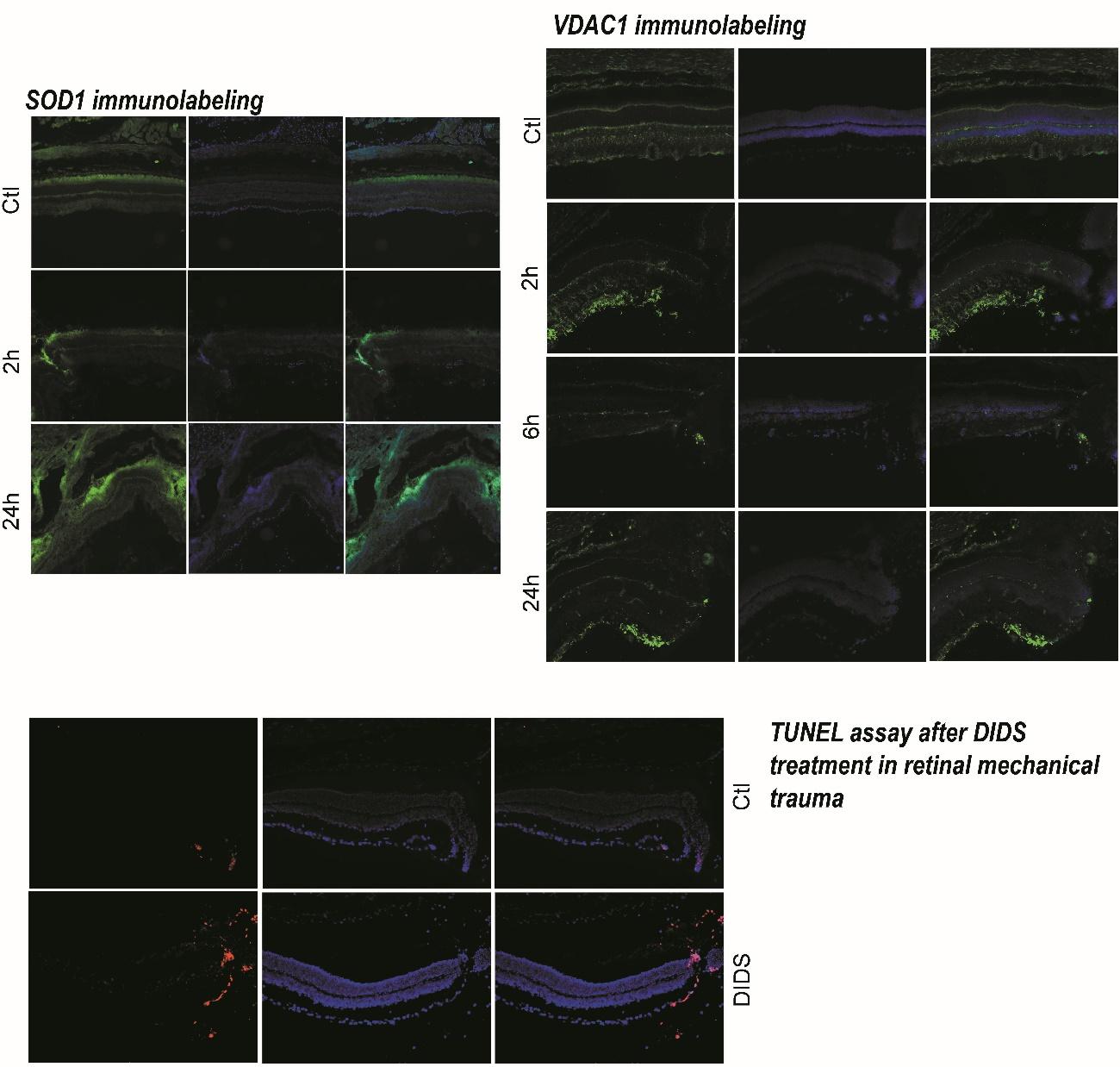


Lastly, figure 5 indicates the cellular fractionation technique validation using antibodies for mitochondria, plasma membrane, and endoplasmic reticulum.

Figure 5:


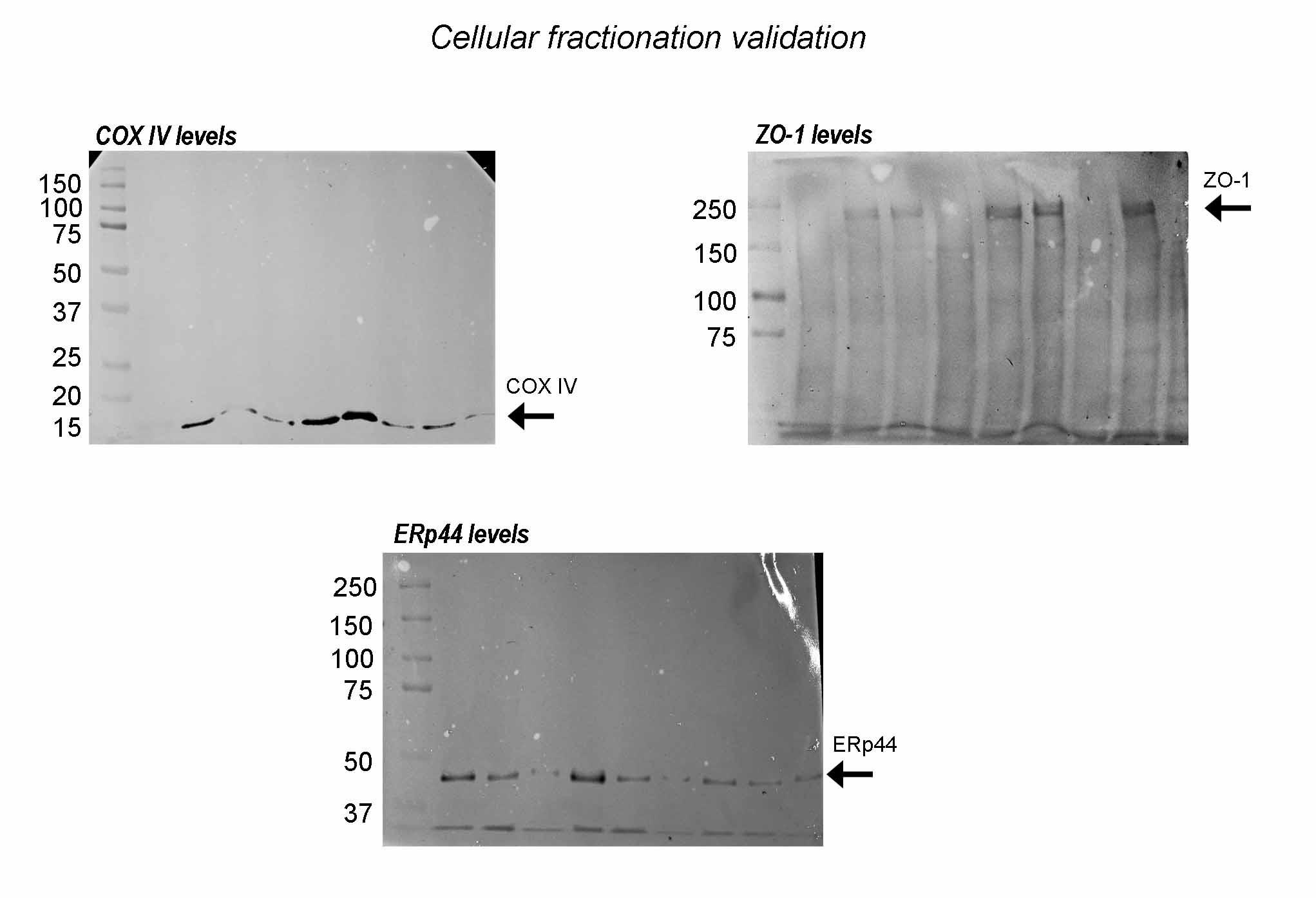

Supplement: Supplementary file 2 — Supplemental material [file 41419_2022_4755_MOESM2_ESM.docx]
